# Three New Records of Pathogens Causing Stem Blight on *Vaccinium corymbosum* in China

**DOI:** 10.3390/plants14050647

**Published:** 2025-02-20

**Authors:** Yueyan Zhou, Linna Wu, Kaixuan Ren, Meng Wang, Nannan Wang, Khanobporn Tangtrakulwanich, Xinghong Li, Kandawatte Wedaralalage Thilini Chethana, Kevin D. Hyde, Wei Zhang, Jiye Yan

**Affiliations:** 1 School of Science, Mae Fah Luang University, Chiang Rai 57100, Thailand; 2 Beijing Key Laboratory of Environment Friendly Management on Fruit Diseases and Pests in North China, Institute of Plant Protection, Beijing Academy of Agriculture and Forestry Sciences, Beijing 100097, China; 3 Centre of Excellence in Fungal Research, Mae Fah Luang University, Chiang Rai 57100, Thailand

**Keywords:** *Colletotrichum*, *Curvularia*, *Diaporthe*, morphology, phylogeny, Koch’s postulates, new host records

## Abstract

Stem blight is a significant disease affecting blueberries worldwide, caused by various pathogens. This study investigated stem blight disease in Ji’an, Jilin Province, China. Fungi isolated from diseased stems were identified as *Colletotrichum temperatum*, *Curvularia austriaca*, and *Diaporthe unshiuensis* based on morphological characters and multi-locus phylogenetic analyses using the internal transcribed spacer (ITS) region, glyceraldehyde 3-phosphate dehydrogenase (*gapdh*), chitin synthase (*chs*), actin (*act*), β-tubulin (*tub2*), the translation elongation factor 1-alpha (*tef1-α*), calmodulin (*cal*), and histone 3 (*his3*) regions. Pathogenicity test was conducted on detached green blueberry shoots, all shoots inoculated by mycelium plugs presented necrotic lesions with dark brown margins, while the control (PDA plugs) group did not show any symptoms. Koch’s postulates were confirmed by re-isolating the inoculated pathogen from the disease symptoms. The study provides three new host–pathogen records of fungi associated with blueberry stem blight.

## 1. Introduction

Blueberry (*Vaccinium* spp.), well known for its rich bioactive compounds that promote health, is widely recognized as a “super fruit” [1]. The high nutritional and economic value of blueberries has driven rapid development in the blueberry industry. Over the past five years, the global area under cultivation and blueberry yield increased from 198,817 ha and 1.28 million tons to 262,417 ha and 1.78 million tons, respectively [2]. In China, blueberries are now planted in 26 of 34 provinces [3].

Despite the high resilience of blueberries, various diseases inevitably emerge throughout their lifespan, including fruit rot, stem blight, leaf spot, powdery mildew, and root rot, which pose serious threats to plant health [4]. Among these various diseases, stem blight is both common and severe, resulting in reduced production and economic losses [5]. In New Zealand, the disease incidence has reached 18%, causing $500,000 in annual losses [6]; in Chile, the incidence rate ranges from 15 to 45% [7]. As the top producer of blueberries, China also suffered 10% to 25% of crop damage from the disease [8].

Stem blight is caused by several fungal pathogens that infect plants through wounds or natural openings, leading to vascular damage, wood discoloration, and even the death of the plant [5,9]. As a general term, stem blight is also known as stem canker or dieback. Typical symptoms of the disease are reddish-brown to gray-brown lesions on stems, shoots, or twigs [10,11,12]. *Pestalotiopsis*-like (*Pestalotiopsis* and *Neopestalotiopsis*) species, *Diaporthe*, and many genera of Botryosphaeriaceae (*Botryosphaeria*, *Neofusicoccum*, and *Lasiodiplodia*) are major causal agents of stem blight worldwide [9,10,13]. In addition to these groups, *Calonectria*, *Colletotrichum*, *Nigrospora*, and *Sphaerulina* species have been reported to cause stem blight on blueberries [14,15,16,17]. The diversity of pathogens contributing to stem blight highlights its nature as a disease complex, making it difficult to distinguish based on field symptoms. Despite many studies conducted on stem blight, many fungal pathogens affecting blueberries may still be undiscovered. Accurate identification of new host-pathogen relationships is crucial for understanding pathogen characteristics and is essential for effective disease diagnosis and management [18]. During this investigation on blueberry stem blight in Jilin Province, three fungal pathogens were isolated from blueberry and verified via pathogenicity tests. The objective of the current study is to expand knowledge on the newly emerging pathogens associated with stem blight disease and contribute to the research on fungal diversity in blueberries. The study also presents a case of mixed-pathogen infection.

## 2. Results

### 2.1. Field Symptoms and Fungal Isolation

**Figure 1:**
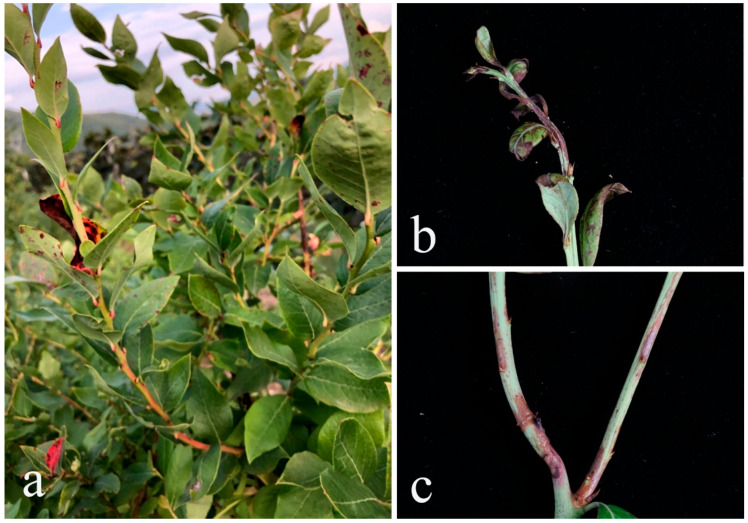
Disease symptoms of blueberry stem blight. (**a**) field symptoms of blueberry stem blight; (**b**) disease symptoms on the tip of the stem; (**c**) disease symptoms on the middle of the stem.

During the investigation on blueberry stem disease in Jilin Province, China, symptomatic blueberry was observed in an orchard under normal management (soil cultivation in an open field) in Ji’an City. Diseased stems showed irregular, reddish-brown lesions that extended to both sides of the stem, and the tip became shriveled and withered. Leaves wilt or exhibit brown spots after 1–2 weeks (Figure 1). Six isolates were obtained from the margin of the lesion of four blueberry samples (variety: ‘Blue Crop’; collection site: annual shoot) and identified as three species: *Colletotrichum temperatum* (JZB330443, JZB330444), *Curvularia austriaca* (JZB3720001, JZB3720002), and *Diaporthe unshiuensis* (JZB320309, JZB320310).

### 2.2. Fungal Identification and Description

*Colletotrichum temperatum*, *Curvularia austriaca*, and *Diaporthe unshiuensis* were identified based on molecular and morphological data. Phylogenetic analyses were conducted for each genus, followed by the corresponding morphological descriptions and taxonomic notes.

#### 2.2.1. *Colletotrichum*

Classification: Glomerellaceae, Glomerellales, Hypocreomycetidae, Sordariomycetes, Ascomycota, Fungi [19]. *Colletotrichum* is the only genus in the Glomerellaceae, with 1075 epithets listed in Index Fungorum [20] and more than 300 accepted species to date [21,22]. The genus includes one of the top 10 important plant pathogens, infecting crops throughout the world and causing significant losses [23].

- Phylogeny

**Figure 2:**
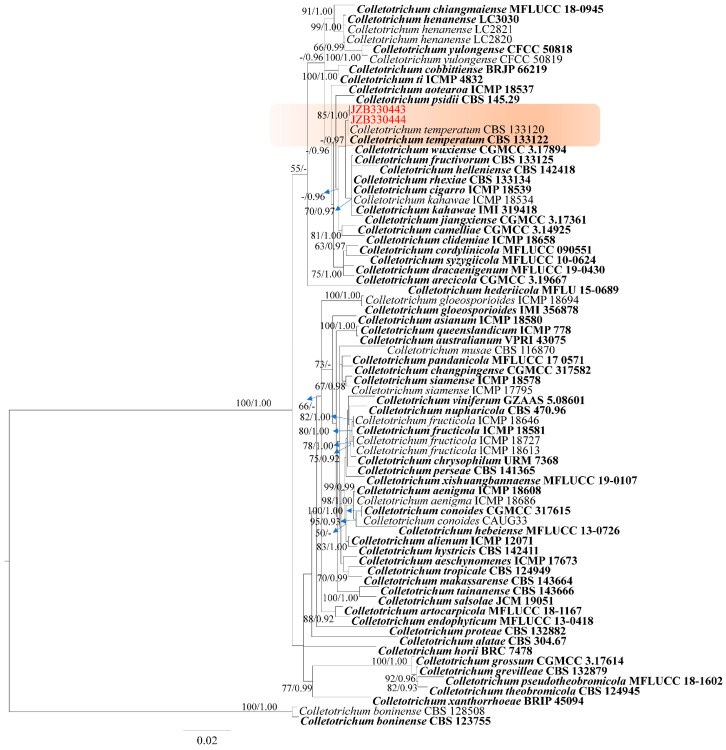
Phylogenetic tree generated by maximum likelihood analysis of the combined ITS, *gapdh*, *chs*, *act*, and *tub* sequence data of species belonging to the *Colletotrichum gloeosporioides* species complex. The tree is rooted with *Colletotrichum boninense* (CBS 123755 and CBS 128508). RAxML bootstrap support values ≥ 50% and Bayesian posterior probabilities ≥ 0.90 (BYPP) are shown near the nodes. The scale bar indicates 0.02 changes. The clade comprising the isolates of the current study is highlighted in orange. The arrowhead points toward the node to which the value belongs. The ex-type strains are in bold, and isolates from the current study are in red.

In the phylogenetic tree of the *Colletotrichum gloeosporioides* species complex, ITS, *gapdh*, *chs*, *act*, and *tub* gene sequences of 70 reference strains, representing 57 taxa, were included [21,24]. The tree is rooted with *Colletotrichum boninense* (CBS 123755 and CBS 128508). The tree topology of the ML analysis was similar to the Bayesian analysis. The best-scoring RAxML tree with a final likelihood value of −9965.268605 is presented (Figure 2). The matrix had 818 distinct alignment patterns, with 9.03% of undetermined characters or gaps. Estimated base frequencies were as follows: A = 0.226449, C = 0.302220, G = 0.238651, T = 0.232680; substitution rates AC = 1.012333, AG = 2.893451, AT = 1.007060, CG = 0.896810, CT = 4.881719, GT = 1.000000; gamma distribution shape parameter α = 1.066665. The tree topology aligns with the recent phylogenies [21]. Two isolates generated in this study clustered with representative strains of *C. temperatum* with 85% ML bootstrap support and 1.00 BYPP (Figure 2).

- Taxonomy

**Figure 3:**
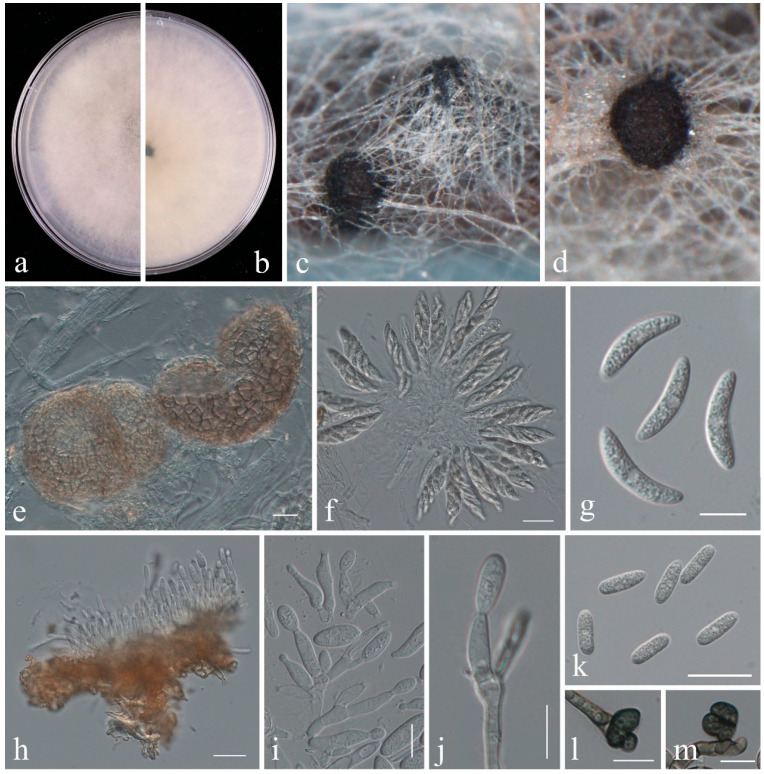
*Colletotrichum temperatum* (JZB330444). (**a**) an upper view of colonies on the PDA; (**b**) reverse view of colonies on the PDA; (**c**–**e**) ascomata; (**f**) asci; (**g**) ascospores; (**h**) section view of acervulus; (**i**,**j**) conidiophores; (**k**) conidia; (**l**,**m**) appressoria. Scale bars: (**e**,**f**,**h**,**k**) =20 μm; (**g**–**j**,**l**,**m**) =10 μm.

***Colletotrichum temperatum*** V.P. Doyle, P.V. Oudem. & S.A. Rehner, PLoS ONE 8(12): e51392, 17 (2013) [25] (Figure 3).

Index Fungorum no: IF 801463; Facesoffungi Number: FoF 16980.

*Pathogenic* on *Vaccinium corymbosum*. **Sexual morph** developed on PNA: *Ascomata* 156–387 × 147–347 μm ($\bar{x}$ = 259.7 × 222.6 μm, n = 15), solitary to clustered, subglobose to obpyriform, dark brown to black. *Asci* 38–64 × 8.5–13 μm ($\bar{x}$= 49.2 × 10.7 μm, n = 30), 8-spored, obclavate, hyaline. *Ascospores* 15–25.5 × 4–5.5 μm ($\bar{x}$= 19.0 × 4.90 μm, n = 30), uniseriate to biseriate, reniform to lunate, aseptate, hyaline, with granular contents. **Asexual morph** developed on the blueberry stem. *Conidiomata* 295–478 × 213–371 μm ($\bar{x}$ = 390.6 × 290.1 μm, n = 15), acervular, semi-immersed, secreting orange conidial masses. Setae not observed. *Conidiophores* are branched, hyaline, septate, and smooth-walled. *Conidiogenous cells* 8–18 × 3–5 μm ($\bar{x}$ = 13.5 × 3.8 μm, n = 30), cylindrical to ampulliform, tapering toward the apex, hyaline. *Conidia* 13.5–17 × 5–6 μm ($\bar{x}$ = 15.4 × 5.4 μm, n = 30), subcylindrical, straight, hyaline, aseptate, often rounded ends, with some having one end rounded and the other slightly acute. *Appressoria* 8–16.5 × 5.5–12.5 μm ($\bar{x}$= 11.5 × 8.1 μm, n = 30), single, terminal, irregular, olivaceous.

Colony Characters—Colonies on PDA reach 85 mm diam. after six days, white to pale gray, flat with floccose aerial mycelium, reverse grayish white.

Material Examined—CHINA, Jilin Province, Ji’an City, on the diseased stem of *V. corymbosum*, 9 September 2023, X.H. Li (dry cultures JZBH330443, JZBH330444), living cultures JZB330443, JZB330444.

Sequence data—JZB330443: ITS: PQ567005 (ITS5/ITS4); *gapdh*: PQ573048 (GDF/GDR); *chs*: PQ573050 (CHS-79F/CHS-345R); *act*: PQ573052 (ACT-512F/783R); *tub*: PQ573054 (T1/Bt2b); JZB330444: ITS: PQ567006 (ITS5/ITS4); *gapdh*: PQ573049 (GDF/GDR); *chs*: PQ573051 (CHS-79F/CHS-345R); *act*: PQ573053 (ACT-512F/783R); *tub*: PQ573055 (T1/Bt2b).

Notes—Isolates generated in this study (JZB330443, JZB330444) clustered with the ex-type of *Colletotrichum temperatum* (CBS 133122) with 85% ML bootstrap support and 1.00 BYPP (Figure 2). Morphologically, the ascospore length of JZB330444 (15–25.5 μm, $\bar{x}$ = 19.0 μm) cultured on PNA (Figure 3) is larger than that of the ex-type (CBS 133122, 14.3–15.3 μm) of *C. temperatum* cultured on CMA, and the conidium width of JZB330444 (5–6 μm, $\overset{¯}{x}$ = 5.4 μm) cultured on PNA is larger than that of the ex-type (CBS 133122, 4.5–4.7 μm) of *C. temperatum* cultured on CMA [25]; the differences may be caused by the difference in the medium. *Colletotrichum temperatum* was introduced by Doyle and colleagues [25] from the decayed fruit of cranberry (*Vaccinium macrocarpon*) in the USA. This species has also been reported to be associated with grapefruit rot in the USA and cherry leaf spot in China [26,27]. This is the first report of *Colletotrichum temperatum* as a pathogen on *V. corymbosum*.

#### 2.2.2. *Curvularia*

Classification: Pleosporaceae, Pleosporales, Pleosporomycetidae, Dothideomycetes, Ascomycota, Fungi [19]. *Curvularia* is the largest genus of helminthosporioid fungi, with 248 species epithets listed in Index Fungorum [20] and 143 accepted species with DNA sequence data [22]. The genus is distributed across the world as pathogens or saprobes with a broad host range, especially a threat to Poaceae crops [28].

- Phylogeny

**Figure 4:**
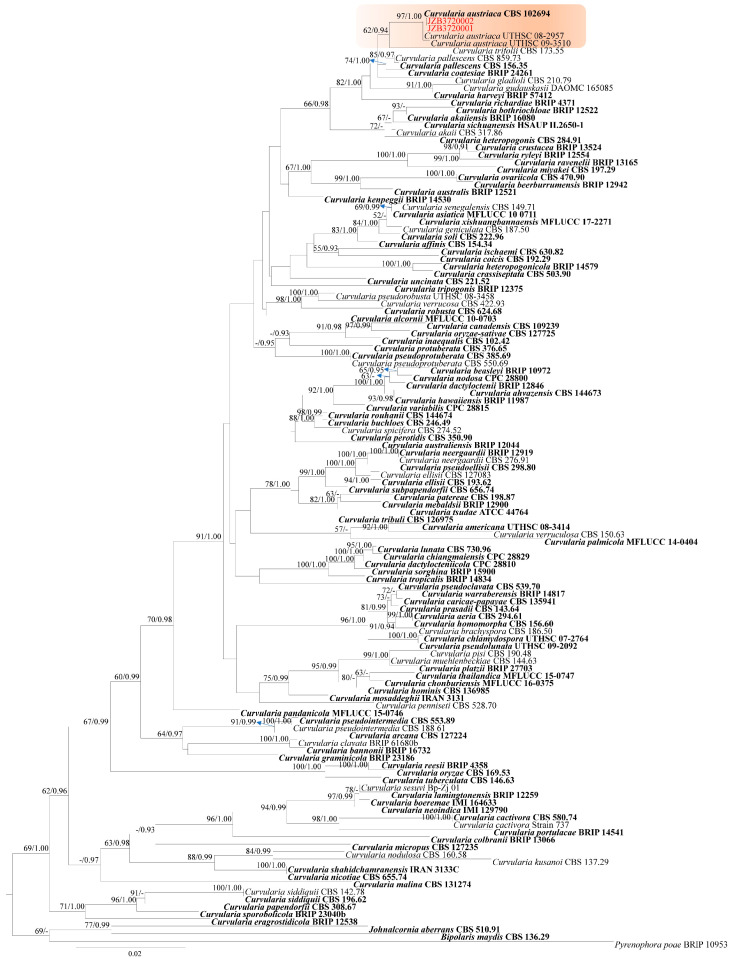
Phylogenetic tree generated by maximum likelihood analysis of the combined ITS, *gapdh*, and *tef* sequence data of species belonging to *Curvularia*. The tree is rooted with *Bipolaris maydis* (CBS 136.29), *Johnalcornia aberrans* (CBS 510.91), and *Pyrenophora poae* (BRIP 10953). RAxML bootstrap support values ≥ 50% and Bayesian posterior probabilities ≥ 0.90 (BYPP) are shown near the nodes. The scale bar indicates 0.02 changes. The clade comprising the isolates of the current study is highlighted in orange. The arrowhead points toward the node to which the value belongs. The ex-type strains are in bold, and isolates from the current study are in red.

The phylogenetic tree of the combined ITS, *gapdh*, and *tef* sequences for *Curvularia* comprised 124 sequences, representing 115 taxa with *Bipolaris maydis* (CBS 136.29), *Johnalcornia aberrans* (CBS 510.91), and *Pyrenophora poae* (BRIP 10953) as the outgroup [28]. The tree topology of the ML analysis was similar to the Bayesian analysis. The best-scoring RAxML tree with a final likelihood value of −14864.109263 is presented (Figure 4). The matrix had 655 distinct alignment patterns, with 17.23% of undetermined characters or gaps. Estimated base frequencies were as follows: A = 0.228223, C = 0.307112, G = 0.244933, T = 0.219732; substitution rates AC = 1.125622, AG = 4.404677, AT = 1.020378, CG = 1.429845, CT = 8.302359, GT = 1.000000; gamma distribution shape parameter α = 0.636951. The topology of the tree is similar to the phylogenies in the previous study [28]. Our two isolates clustered with representative strains of *C. austriaca* with 97% ML bootstrap support and 1.00 BYPP (Figure 4).

- Taxonomy

**Figure 5:**
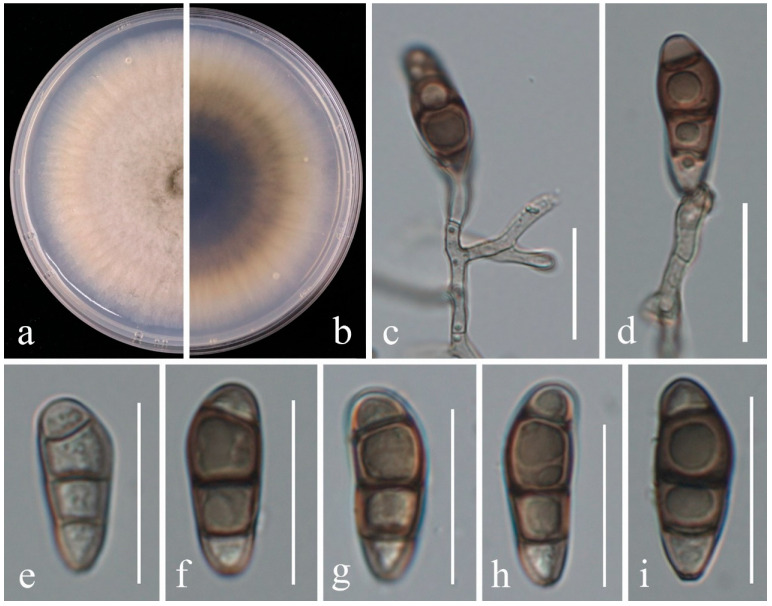
*Curvularia austriaca* (JZB3720002). (**a**) an upper view of colonies on the PDA; (**b**) reverse view of colonies on the PDA; (**c**,**d**) conidiogenous cells and conidia; (**e**–**i**) conidia. Scale bars: (**c**–**i**) =20 μm.

***Curvularia austriaca*** Y. Marín & Crous, in Marin-Felix, Hernández-Restrepo & Crous, Mycol. Progr. 19(6): 564 (2020) [28] (Figure 5).

Index Fungorum no: IF 830045; Facesoffungi Number: FoF 16981; Figure x.

*Pathogenic* on *Vaccinium corymbosum*. **Sexual morph:** Not observed. **Asexual morph:** On PDA: *hyphae* 2–4 μm wide, branched, subhyaline to pale brown, septate. *Conidiophores* are simple, rarely branched, straight to flexuous, often geniculate at the apex, pale brown, and septate. *Conidiogenous cells* 6–14 × 3–5.5 μm ($\bar{x}$ = 10.0 × 4.0 μm, n = 10), terminal or intercalary, subcylindrical to slightly swollen, subhyaline to pale brown, smooth-walled. *Conidia* 21–29.5 × 7.5–12 μm ($\bar{x}$ = 25.5 × 9.7 μm, n = 30), straight or slightly curved, ellipsoidal to obovoid, middle cells slightly enlarged, smooth-walled to finely verruculose, apical and basal cells hyaline to pale brown, middle cells dark brown in matured conidia, 3 distoseptate, middle septa dark brown. *Hila* 1.5–2.5 μm wide, protruding, darkened, thickened. *Chlamydospores* not observed.

Culture characteristics—Colonies on PDA reach 85 mm in diameter after 7 days, margin lobulate, luteous to orange, umber in the center, aerial mycelium moderate, slightly cottony; reverse luteous to orange.

Material Examined—CHINA, Jilin Province, Ji’an City, on the diseased stem of *V. corymbosum*, 9 September 2023, X.H. Li (dry cultures JZBH3720001, JZBH3720002), living cultures JZB3720001, JZB3720002.

Sequence data—Sequence data—JZB3720001: ITS: PQ568977 (ITS5/ITS4); *gapdh*: PQ573056 (gpd1/gpd2); *tef*: PQ573058 (EF1-983F/2218R); JZB3720002: ITS: PQ568978 (ITS5/ITS4); *gapdh*: PQ573057 (gpd1/gpd2); *tef*: PQ573059 (EF1-983F/2218R).

Notes —Two isolates obtained from the blueberry stems in the present study (JZB3720001 and JZBH3720002) phylogenetically clustered with *Curvularia* strains (CBS 102694, UTHSC 08-2957 and UTHSC 09-3510), with 97% ML bootstrap support and 1.00 BYPP (Figure 4). The morphological characters of our isolates (Figure 5) are in accordance with the description of the *C. austriaca* type [28]. This species was introduced by Marin-Felix and colleagues from a human [28], and there are no other records from plant hosts afterwards. This is the first report of *C. austriaca* associated with stem blight on *Vaccinium corymbosum*.

#### 2.2.3. *Diaporthe*

Classification: Diaporthaceae, Diaporthales, Diaporthomycetidae, Sordariomycetes, Ascomycota, Fungi [19]. *Diaporthe* is cosmopolitan with a wide range of plant hosts, and the members in *Diaporthe* are well-known as phytopathogens infecting all parts of the plant, as well as endophytes and saprobes [29]. *Diaporthe* is a huge genus with 1304 epithets listed in Index Fungorum [20]. However, species were previously identified by morphological characters and host association. In recent years, the development of multi-locus phylogenetic analysis promoted the accurate identification of *Diaporthe*, and the taxonomy was revised in a series of studies [30,31,32].

- Phylogeny

**Figure 6:**
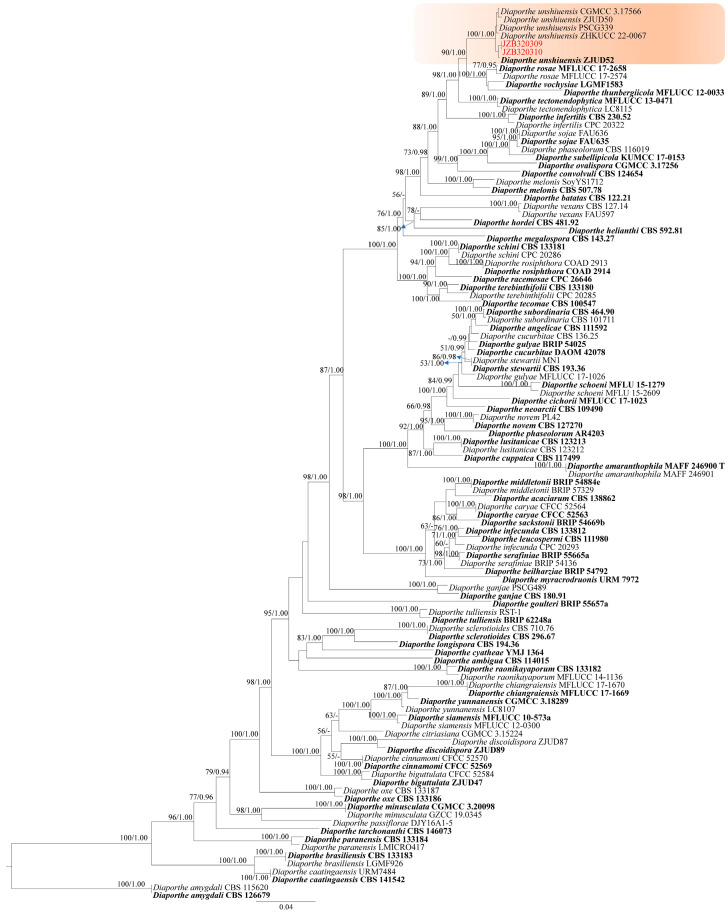
Phylogenetic tree generated by maximum likelihood analysis of combined ITS, *tef*, *tub*, *cal*, and *his* sequence data of species belonging to the *Diaporthe sojae* species complex. The tree is rooted with *D. amygdali* (CBS 126679 and CBS 115620). RAxML bootstrap support values ≥ 50% and Bayesian posterior probabilities ≥ 0.90 (BYPP) are shown near the nodes. The scale bar indicates 0.04 changes. The background color represents the species identified. The arrowhead points toward the node to which the value belongs. The ex-type strains are in bold, and isolates from the current study are in red.

The phylogenetic tree of *Diaporthe* was constructed using five gene sequences (ITS, *tef*, *tub*, *cal*, and *his*), including 108 reference isolates representing 66 taxa in the *Diaporthe sojae* species complex [32]. The tree is rooted with *D. amygdali* isolates (CBS 126679 and CBS 115620). The tree topology of the ML analysis was similar to the Bayesian analysis. The best-scoring RAxML tree with a final likelihood value of −27986.001494 is presented (Figure 6). The matrix had 1208 distinct alignment patterns, with 19.34% of undetermined characters or gaps. Estimated base frequencies were as follows: A = 0.214688, C = 0.319197, G = 0.242643, T = 0.223473; substitution rates AC = 1.222391, AG = 3.869013, AT = 1.482265, CG = 1.021922, CT = 5.320754, GT = 1.000000; gamma distribution shape parameter α = 1.100460. The topology of the tree is consistent with the recent phylogenies of the recent study [32]. Our two isolates clustered with representative strains of *D. unshiuensis* (Figure 6).

- Taxonomy

**Figure 7:**
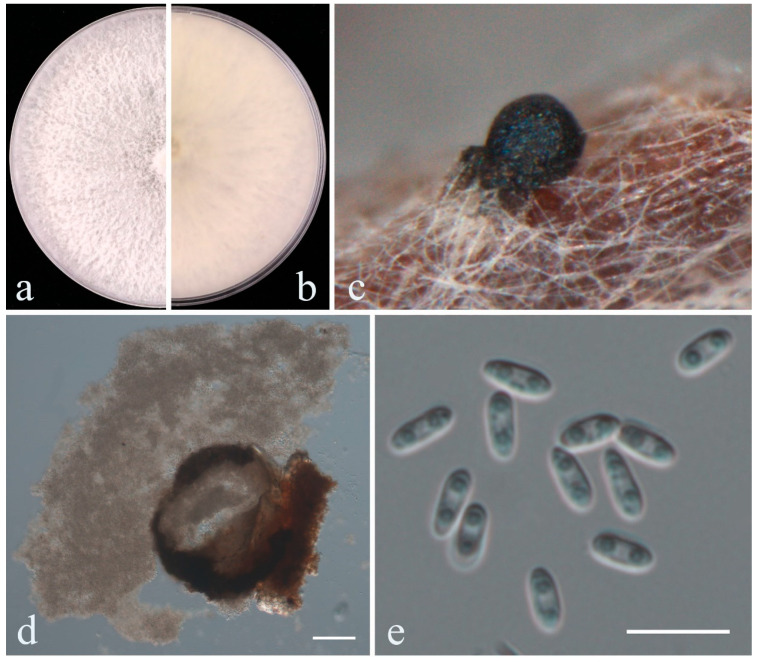
*Diaporthe unshiuensis* (JZB320309). (**a**) an upper view of colonies on the PDA; (**b**) reverse view of colonies on the PDA; (**c**) conidiomata sporulating on PNA; (**d**) cross section of conidiomata; e alpha conidia. Scale bars: (**d**) =100 μm; (**e**) =10 μm.

***Diaporthe unshiuensis*** F. Huang, K.D. Hyde & Hong Y. Li, in Huang, Udayanga, Mei, Fu, Hyde & Li, Fungal Biology 119(5): 344 (2015) [33] (Figure 7).

Index Fungorum no: IF 810845; Facesoffungi Number: FoF 09422.

*Pathogenic* on *Vaccinium corymbosum*. **Sexual morph:** Not observed. **Asexual morph:** On PNA: *Pycnidia* 103–371 × 80–285 μm ($\bar{x}$ = 156.9 × 125.4 μm, n = 25), globose to subglobose, dark brown to black. *Conidiophores* were not observed. *Conidiogenous cells* were not observed. *Alpha conidia* 6–8.5 × 2–3 μm ($\bar{x}$ = 6.7 × 2.7 μm, n = 50), straight to slightly curved, ellipsoidal to fusiform, sometimes with one end obtuse and the other acute, hyaline, smooth, aseptate, biguttulate. *Beta conidia* not observed.

Culture characteristics—Colonies on PDA reach 85 mm in diameter after 4 days, white and turning to gray with aging, reverse white or pale brown pigmentation at the center.

Material Examined—CHINA, Jilin Province, Ji’an City, on the diseased stem of *V. corymbosum*, 9 September 2023, X.H. Li (dry cultures JZBH320309, JZBH320310), living cultures JZB320309, JZB320310.

Sequence data—ITS: PQ569052 (ITS5/ITS4); *tef*: PQ573060 (EF1-688F/1251R); *tub*: PQ573062 (Bt2a/Bt2b); *cal*: PQ573064 (CAL-228F/737R); *his*: PQ573066 (CYLH3F/H3-1b); ITS: PQ569053 (ITS5/ITS4); *tef*: PQ573061 (EF1-688F/1251R); *tub*: PQ573063 (Bt2a/Bt2b); *cal*: PQ573065 (CAL-228F/737R); *his*: PQ573067 (CYLH3F/H3-1b).

Notes—In the phylogenetic analysis, two isolates (JZB320309, JZB320310) obtained from this study formed a well-supported clade with the ex-type strain of *Diaporthe unshiuensis* (ZJUD52), with 100% ML bootstrap support and 1.00 BYPP (Figure 6). Morphologically, the two isolates (Figure 7) are similar to the ex-type of *D. unshiuensis* [33]. *Diaporthe unshiuensis* was first reported by Huang and colleagues [33] as an endophyte on the fruits of *Citrus unshiu* and on asymptomatic branches and twigs of *F. margarita* in China. Subsequently, the species was also isolated from asymptomatic twigs of *Carya illinoensis* [34] and the healthy root of *M. officinalis* [35]. However, later it was found as a pathogen causing shoot blight, canker and dieback on fruit trees, including citrus, pear, kiwifruit, grapevine and peach [36,37,38,39,40]. The species was mostly distributed in China and also reported from soybeans in the USA [41]. This is the first report of *D. unshiuensis* causing stem blight on *Vaccinium corymbosum*.

### 2.3. Pathogenetic Assay

**Figure 8:**
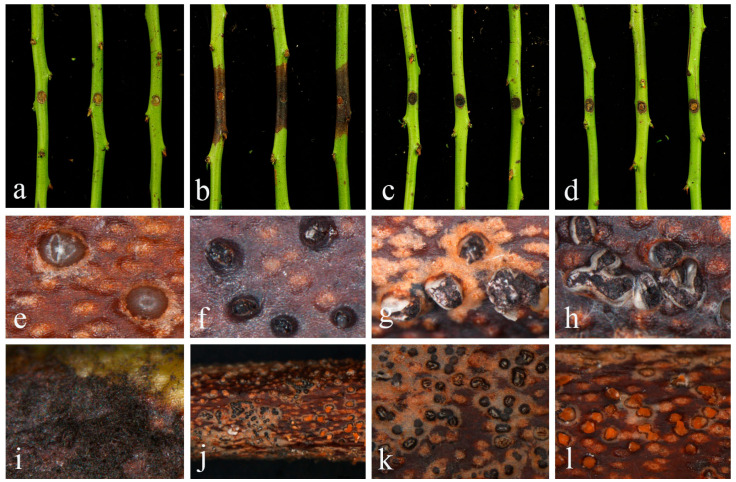
Pathogenicity assay on *Vaccinium corymbosum* shoots (**a**–**d**). (**a**) Control treatment, (**b**) inoculated by *Diaporthe unshiuensis*, (**c**) inoculated by *Curvularia austriaca*, (**d**) inoculated by *Colletotrichum temperatum*, (**e**–**h**) Conidiomata of *Diaporthe unshiuensis* on the shoot lesions, (**i**) mycelia of *Curvularia austriaca* on the shoot lesion, (**j**) Ascomata and conidiomata produced by *Colletotrichum temperatum* on the shoot lesion, (**k**) Ascomata of *Colletotrichum temperatum* on the shoot lesion, (**l**) Conidiomata of *Colletotrichum temperatum* on the shoot lesion.

The virulence of *Colletotrichum temperatum* (JZB330444), *Curvularia austriaca* (JZB3720002), and *Diaporthe unshiuensis* (JZB320309) was assessed using a detached green shoots assay on *Vaccinium corymbosum*. Two weeks post-inoculation, necrotic lesions with dark brown margins were observed on all three treatments (Figure 8). The shoots inoculated with *D. unshiuensis* showed significant lesion development. Reddish-brown lesions extended upwards and downwards from the inoculation point, with the emergence of the brownish conidiomata that became black upon maturation, either solitary or clustered in groups of 3–5 conidiomata. On the shoots inoculated with *Colletotrichum* and *Curvularia*, lesions developed slowly, while dense black mycelia of *Curvularia* and fruiting bodies of *Colletotrichum* appeared at the inoculation point, leading to blight and necrosis of plant tissue. Interestingly, black ascomata and conidiomata secreting conidial masses of *Colletotrichum* appeared simultaneously. Twenty-one days after inoculation, *D. unshiuensis* developed the largest lesions (3.08 cm), while *C. temperatum* and *Curvularia austriaca* were less aggressive, with lesions of 0.75 and 0.65 cm, respectively. No lesions developed on the shoots inoculated with PDA disks. Koch’s postulates were fulfilled by re-isolating original fungi from symptomatic stems.

## 3. Discussion

As an important disease, blueberry stem blight has received serious attention in recent years. In China, Botryosphaeriaceae and Pestalotiopsis-like species have been recognized as major pathogens [9,12]. Reports of *Diaporthe* species as pathogens on blueberries have emerged in recent years. To date, 23 *Diaporthe* species have been reported on blueberry, including *Diaporthe ambigua*, *D. amygdali*, *D. asheicola*, *D. australafricana*, *D. baccae*, *D. crousii*, *D. eres*, *D. foeniculina*, *D. hybrida*, *D. leucospermi*, *D. malorum*, *D. nobilis*, *D. oxe*, *D. passiflorae*, *D. phillipsii*, *D. phoenicicola*, *D. rossmaniae*, *D. rudis*, *D. sojae, D. sterillis*, *D. vaccinii*, *D. vacuae,* and *D. viticola* [10,29,42,43,44,45,46,47]. According to the previous studies, symptoms caused by *Diaporthe* spp. appear as reddish to brown necrotic lesions, followed by the production of numerous black pycnidia [10,42]. In this study, *Diaporthe unshiuensis* was reported on blueberry for the first time, with consistent symptoms observed in the pathogenicity test. Reddish-brown lesions expanded quickly, and black pycnidia formed in the late stage. *Colletotrichum* is also a ubiquitous phytopathogen. Diseases caused by *Colletotrichum*, usually known as anthracnose, lead to extensive yield and economic loss in agricultural production [48]. On blueberry, *Colletotrichum* species, especially *C. acutatum*, *C. gloeosporioides*, and *C. fioriniae*, are major pathogens causing anthracnose fruit rot [49], while *Colletotrichum* species have also been found to cause stem blight in the recent years. *Colletotrichum acutatum*, *C. siamense*, *C. kahawae*, *C. karstii*, *C. nymphaeae*, and *C. sichuaninese* have been reported to cause anthracnose symptoms on blueberry stems, showing dark brown to black spots and wilting [50]. In addition, blueberry fruits affected by anthracnose usually form acervuli exuding pink or salmon conidial mass on the lesion surface in the late stage [49,51]. This symptom was not described on blueberry stem blight in previous studies [15,50]. This study presents the first record of *C. temperatum* on blueberry, and it is interesting that in the pathogenicity test, conidiomata exuding salmon conidial mass was observed, while ascomata in sexual morph also appeared at the same time. *Curvularia* is a filamentous fungus that contains many important plant pathogens causing leaf spots, blight, root rot, and other diseases on cereal crops [52,53]. The genus also comprises opportunistic pathogens of humans. However, the genus has not been reported on blueberries previously. *Curvularia austriaca*, identified in this study, was originally reported from humans by Marin-Felix and colleagues [28] and is now reported on blueberry for the first time in this study. The three species given in this study are newly emerging pathogens of blueberry stem blight; therefore, whether they become the dominant pathogen for disease prevalence needs to be further studied. However, for the important position of the three genera, which are important pathogens, we could not neglect their influence on the disease.

Many taxa that occur as endophytes could switch to pathogens under certain conditions, and fungal species initially isolated from healthy plant tissues could also be pathogenic on other hosts [54]. *Diaporthe unshiuenses*, introduced as an endophyte in *Citrus* [33], has been confirmed as a pathogen causing blight, canker, or dieback on fruit trees, developing reddish-brown necrotic lesions on the stem, branch, or shoot [37,40]. In this study, the species was confirmed to be pathogenic in blueberry, suggesting that it may become an emerging prevalent pathogen in blueberries. However, the other two species, *Colletotrichum temperatum* and *Curvularia austriaca*, showed weak virulence on blueberry in the pathogenicity test, indicating that they may not be the major pathogens of stem blight. Nevertheless, their presence cannot be neglected as they may co-infect with *Diaporthe* on blueberry. Interspecies interactions of pathogenic microorganisms may enhance their impact on hosts [55]. Additionally, the same species may exhibit different virulence on different host varieties [56]. The pathogenicity of the above species among blueberry cultivars and the interplay between fungal species need to be further studied. In conclusion, the current study accurately identified stem blight pathogens of blueberry in Ji’an, Jilin Province, China, and provided a case study on emerging and latent pathogens.

## 4. Materials and Methods

### 4.1. Fungal Isolation and Morphology Characterization

In September 2023 (Post-Harvest Growth), stem blight disease was investigated, and diseased samples were collected from a blueberry orchard in Ji’an, Jilin province (N 40°, E 125°). Diseased samples were cut into small pieces, immersed in 75% ethyl alcohol for 30 s and 2% sodium hypochlorite for 2 min successively for surface sterilization, and then washed three times using sterile water. After drying on sterile filter paper, four pieces of tissue were placed on each PDA plate and incubated at 25 °C. Growing colonies were checked after 5–7 days, and small pieces of colony margins were transferred to new PDA plates, waiting for sporulation. Spore suspension was spread on water agar, and germinated single spores were cultured on PDA plates to obtain pure cultures for identification and strain preservation. Spores and sporogenous structures were recorded using ZEN Pro 2012 of the Axio Imager Z2 photographic microscope (Carl Zeiss Microscopy, Oberkochen, Germany). The current study followed the polyphasic approach suggested for fungal identifications [56,57].

### 4.2. DNA Extraction and PCR Amplification

Genome DNA was extracted from 6-day-old mycelium using the TIANcombi DNA Lyse and Det PCR Kit (parameters followed the protocol in the manual of TIANcombi DNA Lyse & Det PCR Kit, Tiangen Biotech Co., Ltd., Beijing, China) and amplified using the C1000 TouchTM thermal cycler (Bio-Rad Laboratories Inc., Hercules, CA, USA) with the following procedure: initial denaturation for 2 min at 98 °C, followed by 34 cycles of denaturation for 10 s at 98 °C and 15 s of annealing and 1 min elongation at 72 °C, and a final extension for 5 min at 72 °C. The PCR solution mixture was composed of 45 μL of Golden Star T6 Super PCR mix (1.1×) (Tsingke Biotechnology Co., Ltd., Beijing, China), 2 μL of forward and reverse primer, respectively, and 1 μL of genomic DNA. The PCR products were visualized on a 1.2% agarose gel stained with ethidium bromide under UV light using a GelDocXR+ Molecular Imager (Bio-Rad, Hercules, CA, USA). Sequencing of PCR products was carried out at the Sino Geno Max Co., Ltd., Beijing, China, using the Sanger sequencing method. Resulting sequences of the internal transcribed spacer (ITS) region were compared with sequences available in the GenBank at the National Center for Biotechnology Information (NCBI) using the Basic Local Alignment Search Tool (BLAST) tool (https://blast.ncbi.nlm.nih.gov/Blast.cgi, accessed on 28 August 2024). Other gene loci were then amplified according to the resulting genus. For *Colletotrichum* species, ITS, *gapdh*, *chs*, *act*, and *tub* gene regions were amplified; for *Curvularia* species, ITS, *gapdh*, and *tef* gene regions were amplified, while ITS, *tef*, *tub*, *cal*, and *his* gene regions were amplified for *Diaporthe* species. Primer information for each locus is listed in Table 1. Sequence data produced in this study were deposited in the GenBank (Supplementary Table S1).

### 4.3. Phylogenetic Analyses

Newly produced sequences were searched in the BLASTn tool and preliminarily identified at the genus level. Reference sequences (*Colletotrichum*: ITS, *gapdh*, *chs*, *act*, and *tub* regions; *Curvularia*: ITS, *gapdh*, and *tef* regions; *Diaporthe*: ITS, *tef*, *tub*, *cal*, and *his* regions) were downloaded based on the Blastn results and previous studies. Representative strains, including the ex-type of each species of *Curvularia*, were used for analyses, and for *Colletotrichum* and *Diaporthe*, representative strains from each species complex or section were selected to make preliminary analyses to estimate the species complex or section of the isolates obtained in this study. Newly produced sequences were aligned with reference sequences using MAFFT version 7 (http://mafft.cbrc.jp/alignment/server/, accessed on 28 August 2024) [66] and adjusted manually using BioEdit v7.0.9.0 [67]. Automatic alignments of the datasets were trimmed using trimAl v1.2 [68]. Aligned gene regions were concatenated for the analysis using BioEdit v7.0.9.0.

Maximum likelihood (ML) analysis was conducted with the tool RAxML-HPC2 on ACCESS (8.2.12) [69,70] in the CIPRES Science Gateway (https://www.phylo.org/portal2, accessed on 28 August 2024) [71], using the GTR + I + G model with 1000 non-parametric bootstrapping iterations. The Bayesian Inference (BI) analyses were performed using MrBayes v3.2.7 [72], and posterior probabilities (PP) were determined by Markov Chain Monte Carlo sampling (BMCMC). Six simultaneous Markov chains were run for 2,000,000 generations, and trees were sampled at every 1000th generation until the standard deviation of split frequencies reached 0.01. From the 10,000 trees obtained, the first 2000 representing the burn-in phase of the analyses were discarded, and the remaining 8000 trees were used to calculate PPs in the majority rule consensus tree. Phylogenetic trees were visualized with FigTree v1.4.0 [73] and edited with Microsoft Office PowerPoint 2021 and Adobe Illustrator 2020. The sequences generated in this study were deposited in GenBank, and accession numbers were obtained (Supplementary Table S1).

### 4.4. Pathogenicity Tests

Pathogenicity tests were conducted on one-year-old green shoots of blueberry cv. ‘Duke’ (*Vaccinium corymbosum*). One isolate from each of the three species was selected and inoculated onto fifteen shoots, respectively. Mycelium plugs (4 mm) were made by a disinfected hole puncher from the margin of a 5-day-old colony. The green shoots were cut to 30 cm in length and surface sterilized using 75% ethyl alcohol. Wounds with a 4 mm diameter and 2 mm depth were created using a hole puncher on the internode. Mycelium plugs were placed on the wounds and wrapped with sealing film. PDA plugs were used as control. Inoculated green shoots were inserted into small pots with moist soil and incubated in the greenhouse at 25 °C and 100% humidity; after 48 h, the sealing film was removed, and the humidity was maintained at 80%. Inoculation results were observed after three weeks, and lesions were photographed and measured. Koch’s postulate was confirmed by re-isolating the inoculated fungus, which was identified based on morphological characters.

## Figures and Tables

**Table 1:** Gene regions and primer pairs used in the study.

Gene	Primer	Annealing Temperature (°C)	References
*act*	ACT-512F/783R	59	[58]
ITS	ITS5/ITS4	58	[59]
*chs*	CHS-79F/345R	58	[58]
*gapdh*	gpd1/gpd2	56	[60]
	GDF/GDR	59	[61]
*his*	CYLH3F/H3-1b	58	[62]
*tef*	EF1-688F/1251R	54	[63]
	EF1-983F/2218R	54	[64]
*tub*	T1/Bt2b	58	[62,65]
	Bt2a/Bt2b	58	[62]

## Data Availability

All the sequence data generated in this study are available in GenBank (https://www.ncbi.nlm.nih.gov/nucleotide/, accessed on 28 August 2024), and the accession numbers are stated in the article.

## Supplementary Materials

The following supporting information can be downloaded at https://www.mdpi.com/article/10.3390/plants14050647/s1, Table S1: GenBank accession numbers of the isolates generated in the present study.

## References

[1] Kalt W., Cassidy A., Howard L.R., Krikorian R., Stull A.J., Tremblay F., Zamora-Ros R. (2020). Recent research on the health benefits of blueberries and their anthocyanins. Adv. Nutr..

[2] International Blueberry Organization Global State of the Blueberry Industry Report 2024. https://www.internationalblueberry.org/2024-report/.

[3] U.S. Department of Agriculture China: Blueberry Annual Voluntary 2023. https://fas.usda.gov/data/china-blueberry-annual-voluntary-2023.

[4] Polashock J.J., Caruso F.L., Averill A.L., Schilder A.C. (2017). Compendium of Blueberry, Cranberry, and Lingonberry Diseases and Pests.

[5] Martino I., Lione G., Garbelotto M., Gonthier P., Guarnaccia V. (2024). Modeling the effect of temperature on the severity of blueberry stem blight and dieback with a focus on *Neofusicoccum parvum* and cultivar susceptibility. Horticulturae.

[6] Sammonds J., Billones R., Ridgway H.J., Walter M., Jaspers M.V. (2009). Survey of blueberry farms for Botryosphaeria dieback and crown rot pathogens. N. Z. Plant Prot..

[7] Espinoza J.G., Briceño E.X., Chávez E.R., Úrbez-Torres J.R., Latorre B.A. (2009). *Neofusicoccum* spp. associated with stem canker and dieback of blueberry in Chile. Plant Dis..

[8] Zhao L., Wang Y., He W., Zhang Y. (2019). Stem blight of blueberry caused by *Lasiodiplodia vaccinii* sp. nov. in China. Plant Dis..

[9] Ru S., Ding S., Oliver J.E., Amodu A. (2023). A review of Botryosphaeria stem blight disease of blueberry from the perspective of plant breeding. Agriculture.

[10] Hilário S., Santos L., Alves A. (2021). Diversity and pathogenicity of *Diaporthe* species revealed from a survey of blueberry orchards in Portugal. Agriculture.

[11] Zhao L., Sun W., Zhao L., Zhang L., Yin Y., Zhang Y. (2022). *Neofusicoccum vaccinii*: A novel species causing stem blight and dieback of blueberries in China. Plant Dis..

[12] Zheng X., Liu X., Li X., Quan C., Li P., Chang X., Gu J., Khaskheli M.I., Gong G. (2023). *Pestalotiopsis* species associated with blueberry leaf spots and stem cankers in Sichuan Province of China. Plant Dis..

[13] Santos J., Hilário S., Pinto G., Alves A. (2022). Diversity and pathogenicity of pestalotioid fungi associated with blueberry plants in portugal, with description of three novel species of *Neopestalotiopsis*. Eur. J. Plant. Pathol..

[14] Wright E.R., Folgado M., Rivera M.C., Crelier A., Vasquez P., Lopez S.E. (2008). *Nigrospora sphaerica* causing leaf spot and twig and shoot blight on blueberry: A new host of the pathogen. Plant Dis..

[15] Xu C.N., Zhou Z.S., Wu Y.X., Chi F.M., Ji Z.R., Zhang H.J. (2013). First report of *Colletotrichum acutatum* associated with stem blight of blueberry plants in China. Plant Dis..

[16] Ali S., Hildebrand P.D., Renderos W.E., Abbasi P.A. (2021). Identification and characterization of *Sphaerulina vaccinii* sp. nov. as the cause of leaf spot and stem canker in lowbush blueberry and its epidemiology. Phytopathology.

[17] Chen C., Liang X., Lin Y., Hsiang T., Xiang M., Zhang Y. (2023). First report of leaf spot and stem blight on blueberry (*Vaccinium corymbosum* ‘Bluerain’) caused by *Calonectria pseudoreteaudii* in China. Plant Dis..

[18] Dugan F., Glawe D., Attanayake R., Chen W. (2009). The importance of reporting new host-fungus records for ornamental and regional crops. Plant Health. Prog..

[19] Hyde K.D., Noorabadi M.T., Thiyagaraja V., He M.Q., Johnston P.R., Wijesinghe S.N., Armand A., Biketova A.Y., Chethana K.W.T., Erdoğdu M. (2024). The 2024 outline of fungi and fungus-like taxa. Mycosphere.

[20] Index Fungorum. https://www.indexfungorum.org/Names/Names.asp.

[21] Liu F., Ma Z.Y., Hou L.W., Diao Y.Z., Wu W.P., Damm U., Song S., Cai L. (2022). Updating species diversity of *Colletotrichum*, with a phylogenomic overview. Stud. Mycol..

[22] Bhunjun C.S., Chen Y.J., Phukhamsakda C., Boekhout T., Groenewald J.Z., McKenzi E.H.C., Francisco E.C., Frisvad J.C., Groenewald M., Hurdeal V.G. (2024). What are the 100 most cited fungal genera?. Stud. Mycol..

[23] Dean R., Van Kan J.A.L., Pretorius Z.A., Hammond-Kosack K.E., Di Pietro A., Spanu P.D., Rudd J.J., Dickman M., Kahmann R., Ellis J. (2012). The top 10 fungal pathogens in molecular plant pathology. Mol. Plant Pathol..

[24] Fu M., Crous P.W., Bai Q., Zhang P.F., Xiang J., Guo Y.S., Zhao F.F., Yang M.M., Hong N., Xu W.X. (2019). *Colletotrichum* species associated with anthracnose of *Pyrus* spp. in China. Persoonia.

[25] Doyle V.P., Oudemans P.V., Rehner S.A., Litt A. (2013). Habitat and host indicate lineage identity in *Colletotrichum gloeosporioides* s.l. from wild and agricultural landscapes in north america. PLoS ONE.

[26] Cosseboom S.D., Hu M. (2022). Ontogenic susceptibility of grapevine clusters to ripe rot, caused by the *Colletotrichum acutatum* and *C. gloeosporioides* species complexes. Phytopathology.

[27] Zhou Y.Y., Zhang W., Li Y.M., Ji S.X., Li X.H., Hyde K.D., Zhang K.C., Phillips A.J.L., Manawasinghe I.S., Yan J.Y. (2023). Identification and characterization of *Colletotrichum* species associated with cherry leaf spot disease in China. Plant Dis..

[28] Marin-Felix Y., Hernández-Restrepo M., Crous P.W. (2020). Multi-locus phylogeny of the genus *Curvularia* and description of ten new species. Mycol. Prog..

[29] Gomes R.R., Glienke C., Videira S.I.R., Lombard L., Groenewald J.Z., Crous P.W. (2013). *Diaporthe*: A genus of endophytic, saprobic and plant pathogenic fungi. Persoonia.

[30] Norphanphoun C., Gentekaki E., Hongsanan S., Jayawardena R., Senanayake I., Manawasinghe I., Abeywickrama P., Bhunjun C., Hyde K. (2022). *Diaporthe*: Formalizing the species-group concept. Mycosphere.

[31] Hongsanan S., Norphanphoun C., Senanayake I.C., Jayawardena R.S., Manawasinghe I.S., Abeywickrama P.D., Khuna S., Suwannarach N., Senwanna C., Monkai J. (2023). Annotated notes on *Diaporthe* species. Mycosphere.

[32] Dissanayake A.J., Zhu J.T., Chen Y.Y., Maharachchikumbura S.S.N., Hyde K.D., Liu J.K. (2024). A re-evaluation of *Diaporthe*: Refining the boundaries of species and species complexes. Fungal Divers..

[33] Huang F., Udayanga D., Wang X.H., Hou X., Mei X.F., Fu Y.S., Hyde K.D., Li H.Y. (2015). Endophytic *Diaporthe* associated with *Citrus*: A phylogenetic reassessment with seven new species from China. Fungal Biol..

[34] Yang Q., Fan X.L., Guarnaccia V., Tian C.M. (2018). High diversity of *Diaporthe* species associated with dieback diseases in China, with twelve new species described. Mycokeys.

[35] Luo M., Guo W., Zhao M.P., Manawasinghe I.S., Guarnaccia V., Liu J.W., Hyde K.D., Dong Z.Y., You C.P. (2022). Endophytic *Diaporthe* associated with *Morinda officinalis* in China. J. Fungi.

[36] Manawasinghe I.S., Dissanayake A.J., Li X.H., Liu M., Wanasinghe D.N., Xu J.P., Zhao W.S., Zhang W., Zhou Y.Y., Hyde K.D. (2019). High genetic diversity and species complexity of *Diaporthe* associated with grapevine dieback in China. Front. Microbiol..

[37] Guo Y.S., Crous P.W., Bai Q., Fu M., Yang M.M., Wang X.H., Du Y.M., Hong N., Xu W.X., Wang G.P. (2020). High diversity of *Diaporthe* species associated with pear shoot canker in China. Persoonia.

[38] Du Y.M., Wang X.H., Guo Y.S., Xiao F., Peng Y.H., Hong N., Wang G.P. (2021). Biological and molecular characterization of seven *Diaporthe* species associated with kiwifruit shoot blight and leaf spot in China. Phytopathol. Mediterr..

[39] Wang X.H., Guo Y.S., Du Y.M., Yang Z.L., Huang X.Z., Hong N., Xu W.X., Wang G.P. (2021). Characterization of *Diaporthe* species associated with peach constriction canker, with two novel species from China. MycoKeys.

[40] Xiao X.E., Liu Y.D., Zheng F., Xiong T., Zeng Y.T., Wang W., Zheng X.L., Wu Q., Xu J.P., Crous P.W. (2023). High species diversity in *Diaporthe* associated with *Citrus* diseases in China. Persoonia.

[41] Petrović K., Skaltsas D., Castlebury L.A., Kontz B., Allen T.W., Chilvers M.I., Gregory N., Kelly H.M., Koehler A.M., Kleczewski N.M. (2021). Diaporthe seed decay of soybean [*Glycine Max* (L.) Merr.] is endemic in the United States, but new fungi are involved. Plant Dis..

[42] Elfar K., Torres R., Díaz G.A., Latorre B.A. (2013). Characterization of *Diaporthe australafricana* and *Diaporthe* spp. associated with stem canker of blueberry in Chile. Plant Dis..

[43] Lombard L., van Leeuwen G.C.M., Guarnaccia V., Polizzi G., van Rijswick P.C.J., Rosendahl K.C.H.M., Gabler J., Crous P.W. (2014). *Diaporthe* species associated with *Vaccinium*, with specific reference to Europe. Phytopathol. Mediterr..

[44] Sessa L., Abreo E., Lupo S. (2018). Diversity of fungal latent pathogens and true endophytes associated with fruit trees in Uruguay. J. Phytopatho..

[45] Yu C., Wu C., Li G., Wang C. (2018). First report of *Diaporthe nobilis* causing postharvest rot of blueberry in Shandong Province, China. Plant Dis..

[46] Hilário S., Amaral I.A., Gonçalves M.F.M., Lopes A., Santos L., Alves A. (2020). *Diaporthe* species associated with twig blight and dieback of *Vaccinium Corymbosum* in Portugal, with description of four new species. Mycologia.

[47] Li Y.K., Hu Y., Wu Q.X., Wu Y.Y., Zhang Z.L., Yi R., Song X.H. (2023). First report of *Diaporthe sojae* causing stem canker on blueberry in China. Plant Dis..

[48] Tsushima A., Shirasu K. (2022). Genomic resources of *Colletotrichum* fungi: Development and application. J. Gen. Plant. Pathol..

[49] Neugebauer K.A., Mattupalli C., Hu M., Oliver J.E., VanderWeide J., Lu Y., Sullivan K., Stockwell V.O., Oudemans P., Miles T.D. (2024). Managing fruit rot diseases of *Vaccinium corymbosum*. Front. Plant Sci..

[50] Liu X., Zheng X.J., Khaskheli M.I., Sun X.F., Chang X.L., Gong G.S. (2020). Identification of *Colletotrichum* species associated with blueberry anthracnose in Sichuan, China. Pathogens.

[51] Bell S.R., Hernández Montiel L.G., González Estrada R.R., Gutiérrez Martínez P. (2021). Main diseases in postharvest blueberries, conventional and eco-friendly control methods: A review. LWT.

[52] Kusai N.A., Mior Zakuan Azmi M., Zulkifly S., Yusof M.T., Mohd Zainudin N.A.I. (2016). Morphological and molecular characterization of *Curvularia* and related species associated with leaf spot disease of rice in Peninsular Malaysia. Rend. Fis. Acc. Lincei.

[53] Ram D., Devi T.P., Koti P.S., Jeevan B., Kamil D., Vanapalli C.S., Raghu S., Sunani S.K., Kashyap A.S. (2024). Exploring the taxonomic classification of *Curvularia* genera: Enhancing understanding of phytopathogenic species in Poaceae through morphological and molecular approaches. J. Plant Pathol..

[54] Bhunjun C.S., Phukhamsakda C., Hyde K.D., McKenzie E.H.C., Saxena R.K., Li Q.R. (2024). Do all fungi have ancestors with endophytic lifestyles?. Fungal Divers..

[55] Lamichhane J.R., Venturi V. (2015). Synergisms between microbial pathogens in plant disease complexes: A growing trend. Front. Plant Sci..

[56] Manawasinghe I.S., Phillips A.J.L., Xu J., Balasuriya A., Hyde K.D., Stępień Ł., Harischandra D.L., Karunarathna A., Yan J.Y., Weerasinghe J. (2021). Defining a species in fungal plant pathology: Beyond the species level. Fungal Divers..

[57] Chethana K.W.T., Manawasinghe I.S., Hurdeal V.G., Bhunjun C.S., Appadoo M.A., Gentekaki E., Raspé O., Promputtha I., Hyde K.D. (2021). What are fungal species and how to delineate them?. Fungal Divers..

[58] Carbone I., Kohn L.M. (1999). A method for designing primer sets for speciation studies in filamentous ascomycetes. Mycologia.

[59] White T.J., Bruns T., Lee S., Taylor J., Innis M.A., Gelfand D.H., Sninsky J.J., White T.J. (1990). Amplification and direct sequencing of fungal ribosomal RNA genes for phylogenetics. PCR Protocols: A Guide to Methods and Applications.

[60] Myllys L., Stenroos S., Thell A. (2002). New genes for phylogenetic studies of lichenized fungi: Glyceraldehyde-3-phosphate dehydrogenase and beta-tubulin genes. The Lichenologist.

[61] Guerber J.C., Liu B., Correll J.C., Johnston P.R. (2003). Characterization of diversity in *Colletotrichum acutatum* sensu lato by sequence analysis of two gene introns, mtDNA and intron RFLPs, and mating compatibility. Mycologia.

[62] Glass N.L., Donaldson G.C. (1995). Development of primer sets designed for use with the PCR to amplify conserved genes from filamentous ascomycetes. Appl. Environ. Microbiol..

[63] Alves A., Crous P.W., Correia A., Phillips A.J.L. (2008). Morphological and molecular data reveal cryptic species in *Lasiodiplodia theobromae*. Fungal Diver..

[64] Rehner S.A., Buckley E. (2005). A *Beauveria* phylogeny inferred from nuclear ITS and EF1-a sequences: Evidence for cryptic diversification and links to *Cordyceps* teleomorphs. Mycologia.

[65] O’Donnell K., Cigelnik E. (1997). Two divergent intragenomic rDNA ITS2 types within a monophyletic lineage of the *Fusarium* are nonorthologous. Mol. Phylogenet. and Evol..

[66] Katoh K., Rozewicki J., Yamada K.D. (2019). MAFFT online service: Multiple sequence alignment, interactive sequence choice and visualization. Brief Bioinform..

[67] Hall T.A. (1999). BioEdit: A user-friendly biological sequence alignment editor and analysis program for Windows 95/98/NT. Nucleic Acids Symp. Ser..

[68] Capella-Gutiérrez S., Silla-Martínez J.M., Gabaldón T. (2009). trimAl: A tool for automated alignment trimming in large-scale phylogenetic analyses. Bioinformatics.

[69] Stamatakis A., Hoover P., Rougemont J. (2008). A rapid bootstrap algorithm for the RAxML web servers. Syst. Biol..

[70] Stamatakis A. (2014). RAxML version 8: A tool for phylogenetic analysis and post-analysis of large phylogenies. Bioinformatics.

[71] Miller M.A., Pfeiffer W., Schwartz T. Creating the CIPRES Science Gateway for inference of large phylogenetic trees. Proceedings of the 2010 Gateway Computing Environments Workshop (GCE).

[72] Ronquist F., Huelsenbeck J.P. (2003). MrBayes 3: Bayesian phylogenetic inference under mixed models. Bioinformatics.

[73] Rambaut A. (2012). FigTree Version 1.4. http://tree.bio.ed.ac.uk/software/figtree.

