# Supplementary material for: Three New Records of Pathogens Causing Stem Blight on Vaccinium corymbosum in China
Source: Plants (Basel). 2025 Feb 20;14(5):647. doi: 10.3390/plants14050647 (PMC11901845; doi:10.3390/plants14050647)
Supplement: Supplementary file 1 [file plants-14-00647-s001.zip › plants-3409756-supplementary.pdf]

Supplementary

Table S1 GenBank accession numbers of the isolates generated in the present study

**Species**	**Culture collection number**	**ITS**	***gapdh***	***chs***	***act***	***tub***
Colletotrichum aenigma	ICMP 18608 T	JX010244	JX010044	JX009774	JX009443	JX010389
C. aenigma	ICMP 18686	JX010243	JX009913	JX009789	JX009519	JX010390
C. aeschynomenes	ICMP 17673 T	JX010176	JX009930	JX009799	JX009483	JX010392
C. alatae	CBS 304.67 T	JX010190	JX009990	JX009837	JX009471	JX010383
C. alienum	ICMP 12071 T	JX010251	JX010028	JX009882	JX009572	JX010411
C. aotearoa	ICMP 18537 T	JX010205	JX010005	JX009853	JX009564	JX010420
C. arecicola	CGMCC 3.19667 T	MK914635	MK935455	MK935541	MK935374	MK935498
C. artocarpicola	MFLUCC 18-1167 T	MN415991	MN435568	MN435569	MN435570	MN435567
C. asianum	ICMP 18580 T	FJ972612	JX010053	JX009867	JX009584	JX010406
C. australianum	VPRI 43075 T	MG572138	MG572127	MW091987	MN442109	MG572149
C. camelliae	CGMCC 3.14925 T	KJ955081	KJ954782	MZ799255	KJ954363	KJ955230
C. changpingense	CGMCC 317582 T	KP683152	MZ664048	KP852449	KP683093	MZ673952
C. chiangmaiense	MFLUCC 18-0945 T	MW346499	MW548592	MW623653	MW655578	
C. chrysophilum	URM 7368 T	KX094252	KX094183	KX094083	KX093982	KX094285
C. cigarro	ICMP 18539 T	JX010230	JX009966	JX009800	JX009523	JX010434
C. clidemiae	ICMP 18658 T	JX010265	JX009989	JX009877	JX009537	JX010438
C. cobbittiense	BRJP 66219 T	MH087016	MH094133	MH094135	MH094134	MH094137
C. conoides	CGMCC 3.17615 T	KP890168	KP890162	KP890156	KP890144	KP890174
C. conoides	CAUG33	KP890169	KP890163	KP890157	KP890145	KP890175
C. cordylinicola	MFLUCC 090551 T	JX010226	JX009975	JX009864	HM470235	JX010440
C. dracaenigenum	MFLUCC 19-0430 T	MN921250	MT215577	MT215575	MT313686	

C. endophyticum	MFLUCC 13-0418 T	KC633854	KC832854	MZ799261	KF306258	MZ673954
C. fructicola	ICMP 18581 T	JX010165	JX010033	JX009866	FJ907426	JX010405
C. fructicola	ICMP 18613	JX010167	JX009998	JX009772	JX009491	JX010388
C. fructicola	ICMP 18646	JX010173	JX010032	JX009874	JX009581	JX010409
C. fructicola	ICMP 18727	JX010179	JX010035	JX009812	JX009565	JX010394
C. fructivorum	CBS 133125 T	JX145145	MZ664047	MZ799259	MZ664126	JX145196
C. gloeosporioides	IMI 356878 T	JX010152	JX010056	JX009818	JX009531	JX010445
C. gloeosporioides	ICMP 18694	JX010155	JX009980	JX009796	JX009481	
C. grevilleae	CBS 132879 T	KC297078	KC297010	KC296987	KC296941	KC297102
C. grossum	CGMCC3.17614 T	KP890165	KP890159	KP890153	KP890141	KP890171
C. hebeiense	MELUCC 13-0726 T	KF156863	KF377495	KF289008	KF377532	KF288975
C. hederiicola	MFLU 15-0689 T	MN631384		MN635794	MN635795	
C. helleniense	CBS 142418 T	KY856446	KY856270	KY856186	KY856019	KY856528
C. henanense	LC3030 T	KJ955109	KJ954810	MZ799256	KM023257	KJ955257
C. henanense	LC2820	KM610182	KM610178		KM610172	KM610184
C. henanense	LC2821	KM610183	KM610179		KM610173	KM610185
C. horii	BRC 7478 T	GQ329690	GQ329681	JX009752	JX009438	JX010450
C. hystricis	CBS 142411 T	KY856450	KY856274	KY856190	KY856023	KY856532
C. jiangxiense	CGMCC 3.17361 T	KJ955149	KJ954850	MZ799257	KJ954427	OK236389
C. kahawae	IMI 319418 T	JX010231	JX010012	JX009813	JX009452	JX010444
C. kahawae	ICMP 18534	JX010227	JX009904	JX009765	JX009473	JX010427
C. makassarense	CBS 143664 T	MH728812	MH728820	MH805850	MH781480	MH846563
C. musae	CBS 116870	JX010146	JX010050	JX009896	JX009433	HQ596280
C. nupharicola	CBS 470 .96 T	JX010187	JX009972	JX009835	JX009437	JX010398
C. pandanicola	MFLUCC 17-0571 T	MG646967	MG646934	MG646931	MG646938	MG646926

C. perseae	CBS 141365 T	KX620308	KX620242	MZ799260	KX620145	KX620341
C. proteae	CBS 132882 T	KC297079	KC297009	KC296986	KC296940	KC297101
C. pseudotheobromicola	MFLUCC 18-1602 T	MH817395	MH853675	MH853678	MH853681	MH853684
C. psidii	CBS 145.29 T	JX010219	JX009967	JX009901	JX009515	JX010443
C. queenslandicum	ICMP 778 T	JX010276	JX009934	JX009899	JX009447	JX010414
C. rhexiae	CBS 133134 T	JX145128	MZ664046	MZ799258	MZ664127	JX145179
C. salsolae	JCM l9051 T	JX010242	JX009916	JX009863	JX009562	JX010403
C. siamense	ICMP 18578 T	JX010171	JX009924	JX009865	FJ907423	JX010404
C. siamense	ICMP 17795	JX010162	JX010051	JX009805	JX009506	JX010393
C. syzygiicola	MFLUCC 10-0624 T	KF242094	KF242156		KF157801	KF254880
C. tainanense	CBS 143666 T	MH728818	MH728823	MH805845	MH781475	MH846558
C. temperatum	CBS 133122 T	JX145159	MZ664045	MZ799254	MZ664125	JX145211
C. temperatum	CBS 133120	JX145135				JX145186
C. temperatum	JZB330443	PQ567005	PQ573048	PQ573050	PQ573052	PQ573054
C. temperatum	JZB330444	PQ567006	PQ573049	PQ573051	PQ573053	PQ573055
C. theobromicola	CBS 124945 T	JX010294	JX010006	JX009869	JX009444	JX010447
C. ti	ICMP 4832 T	JX010269	JX009952	JX009898	JX009520	JX010442
C. tropicale	CBS 124949 T	JX010264	JX010007	JX009870	JX009489	JX010407
C. *wuxiense*	CGMCC 3.17894 T	KU251591	KU252045	KU251939	KU251672	KU252200
C. xanthorrhoeae	BRIP 45094 T	JX010261	JX009927	JX009823	JX009478	JX010448
C. xishuangbannaense	MFLUCC 19-0107 T	MW346469	MW537586	MW660832	MW652294	
C. yulongense	CFCC 50818 T	MH751507	MK108986	MH793605	MH777394	MK108987
C. yulongense	CFCC 50819	MK108994	MK108992	MK108991	MK108989	MK108993
C. viniferum	GZAAS 5.08601 T	JN412804	JN412798		JN412795	
C. boninense	CBS 123755 T	JQ005153	JQ005240	JQ005327	JQ005501	JQ005588

C. boninense	CBS 128506	JQ005157	JQ005244	JQ005331	JQ005506	JQ005592
**Species**	**Culture collection number**	**ITS**	***gapdh***	***tef***		
Curvularia aeria	CBS 294.61 ^{T}	HE861850	HF565450			
C. affinis	CBS 154.34 ^{T}	KJ909780	KM230401	KM196566		
C. ahvazensis	CBS 144673 ^{T}	KX139029	MG428693	MG428686		
C. akaii	CBS 317.86	KJ909782	KM230402	KM196569		
C. akaiiensis	BRIP 16080 ^{T}	KJ415539	KJ415407	KJ415453		
C. alcornii	MFLUCC 10-0703 ^{T}	JX256420	JX276433	JX266589		
C. americana	UTHSC 08-3414 ^{T}	HE861833	HF565488			
C. arcana	CBS 127224 ^{T}	MN688801	MN688828	MN688855		
C. asiatica	MFLUCC 10-0711 ^{T}	JX256424	JX276436	JX266593		
C. australiensis	BRIP 12044 ^{T}	KJ415540	KJ415406	KJ415452		
C. australis	BRIP 12521 ^{T}	KJ415541	KJ415405	KJ415451		
C. austriaca	CBS 102694 ^{T}	MN688802	MN688829	MN688856		
C. austriaca	UTHSC 08-2957	HE861846	HF565456	–		
C. austriaca	UTHSC 09-3510	HE861847	HF565458	–		
C. austriaca	JZB3720001	PQ568977	PQ573056	PQ573058		
C. austriaca	JZB3720002	PQ568978	PQ573057	PQ573059		
C. bannonii	BRIP 16732 ^{T}	KJ415542	KJ415404	KJ415450		
C. beasleyi	BRIP 10972 ^{T}	MH414892	MH433638	MH433654		
C. beerburrumensis	BRIP 12942 ^{T}	MH414894	MH433634	MH433657		
C. boeremae	IMI 164633 ^{T}	MH414911	MH433641	–		
C. bothriochloae	BRIP 12522 ^{T}	KJ415543	KJ415403	KJ415449		
C. brachyspora	CBS 186.50	KJ922372	KM061784	KM230405		

C. buchloes	CBS 246.49 T	KJ909765	KM061789	KM196588		
C. cactivora	CBS 580.74R	MN688803	MN688830	MN688857		
C. cactivora	Strain 737	HM598679	HM598682	–		
C. canadensis	CBS 109239 T	MN688804	MN688831	MN688858		
C. caricae-papayae	CBS 135941 T	HG778984	HG779146	–		
C. chiangmaiensis	CPC 28829 T	MF490814	MF490836	MF490857		
C. chlamydospora	UTHSC 07-2764 T	HG779021	HG779151	–		
C. chonburiensis	MFLUCC 16-0375 T	MH275055	MH412747	–		
C. clavata	BRIP 61680b	KU552205	KU552167	KU552159		
C. coatesiae	BRIP 24261 T	MH414897	MH433636	MH433659		
C. coicis	CBS 192.29 T	JN192373	JN600962	JN601006		
C. colbranii	BRIP 13066 T	MH414898	MH433642	MH433660		
C. crassiseptata	CBS 503.90 T	LT631310	LT715882	MN688859		
C. crustacea	BRIP 13524 T	KJ415544	KJ415402	KJ415448		
C. dactylocteniicola	CPC 28810 T	MF490815	MF490837	MF490858		
C. dactyloctenii	BRIP 12846 T	KJ415545	KJ415401	KJ415447		
C. ellisii	CBS 193.62 T	JN192375	JN600963	JN601007		
C. ellisii	CBS 127083	MN688805	MN688832	MN688860		
C. eragrostidicola	BRIP 12538 T	MH414899	MH433643	MH433661		
C. geniculata	CBS 187.50	KJ909781	KM083609	KM230410		
C. gladioli	CBS 210.79	HG778987	HG779123	–		
C. graminicola	BRIP 23186 T	JN192376	JN600964	JN601008		
C. gudauskasii	DAOMC 165085	AF071338	–	–		
C. harveyi	BRIP 57412 T	KJ415546	KJ415400	KJ415446		
C. hawaiiensis	BRIP 11987 T	KJ415547	KJ415399	KJ415445		

C. heteropogonicola	BRIP 14579 [T]	KJ415548	KJ415398	KJ415444		
C. heteropogonis	CBS 284.91 [T]	JN192379	JN600969	JN601013		
C. hominis	CBS 136985 [T]	HG779011	HG779106	–		
C. homomorpha	CBS 156.60 [T]	JN192380	JN600970	JN601014		
C. inaequalis	CBS 102.42 [T]	KJ922375	KM061787	KM196574		
C. ischaemi	CBS 630.82 [T]	JX256428	JX276440	–		
C. kenpeggii	BRIP 14530 [T]	MH414900	MH433644	MH433662		
C. kusanoi	CBS 137.29	JN192381	–	JN601016		
C. lamingtonensis	BRIP 12259 [T]	MH414901	MH433645	MH433663		
C. lunata	CBS 730.96 [T]	JX256429	JX276441	JX266596		
C. malina	CBS 131274 [T]	JF812154	KP153179	KR493095		
C. mebaldsii	BRIP 12900 [T]	MH414902	MH433647	MH433664		
C. micropus	CBS 127235 [T]	HE792934	LT715859	–		
C. miyakei	CBS 197.29 [T]	KJ909770	KM083611	KM196568		
C. mosaddeghii	IRAN 3131C [T]	MG846737	MH392155	MH392152		
C. muehlenbeckiae	CBS 144.63 [T]	HG779002	HG779108	–		
C. neergaardii	BRIP 12919 [T]	KJ415550	KJ415397	KJ415443		
C. neergaardii	CBS 276.91	MN688806	MN688833	MN688861		
C. neoindica	IMI 129790 [T]	NR_158450	MH433649	MH433667		
C. nicotiae	CBS 655.74 [T]	KJ415551	KJ415396	KJ415442		
C. nodosa	CPC 28800 [T]	MF490816	MF490838	MF490859		
C. nodulosa	CBS 160.58	JN601033	JN600975	JN601019		
C. oryzae	CBS 169.53 [T]	KP400650	KP645344	KM196590		
C. oryzae-sativae	CBS 127725 [T]	MN688808	MN688835	MN688863		
C. ovariicola	CBS 470.90 [T]	MN688809	MN688836	–		

C. pandanicola	MFLUCC 15-0746 [T]	MH275056	MH412748	MH412763		
C. papendorfii	CBS 308.67 [T]	KJ909774	KM083617	KM196594		
C. pallescens	CBS 156.35 [T]	KJ922380	KM083606	KM196570		
C. pallescens	CBS 859.73	HE861848	HF565455	–		
C. palmicola	MFLUCC 14-0404 [T]	MF621582	–	–		
C. patereae	CBS 198.87 [T]	MN688810	MN688837	MN688864		
C. penniseti	CBS 528.70	MN688811	MN688838	–		
C. perotidis	CBS 350.90 [T]	JN192385	KJ415394	JN601021		
C. pisi	CBS 190.48 [T]	KY905678	KY905690	KY905697		
C. platzii	BRIP 27703b [T]	MH414906	MH433651	MH433669		
C. portulacae	BRIP 14541 [T]	KJ415553	KJ415393	KJ415440		
C. prasadii	CBS 143.64 [T]	KJ922373	KM061785	KM230408		
C. protuberata	CBS 376.65 [T]	KJ922376	KM083605	KM196576		
C. pseudoclavata	CBS 539.70 [T]	MN688817	MN688844	MN688869		
C. pseudoellisii	CBS 298.80 [T]	MN688818	MN688845	MN688870		
C. pseudointermedia	CBS 553.89 [T]	MN688819	MN688846	MN688871		
C. pseudointermedia	CBS 188.61	MN688820	MN688847	MN688872		
C. pseudolunata	UTHSC 09-2092 [T]	HE861842	HF565459	–		
C. pseudoprotuberata	CBS 385.69 [T]	MN688821	MN688848	MN688873		
C. pseudoprotuberata	CBS 550.69	MN688822	MN688849	MN688874		
C. pseudorobusta	UTHSC 08-3458	HE861838	HF565476	–		
C. ravenelii	BRIP 13165 [T]	JN192386	JN600978	JN601024		
C. reesii	BRIP 4358 [T]	MH414907	MH433637	MH433670		
C. richardiae	BRIP 4371 [T]	KJ415555	KJ415391	KJ415438		
C. robusta	CBS 624.68 [T]	KJ909783	KM083613	KM196577		

C. rouhanii	CBS 144674 T	KX139030	MG428694	MG428687		
C. ryleyi	BRIP 12554 T	KJ415556	KJ415390	KJ415437		
C. senegalensis	CBS 149.71	HG779001	HG779128	–		
C. sesuvi	Bp-Zj 01	EF175940	–	–		
C. shahidchamranensis	IRAN 3133C T	MH550084	MH550083	–		
C. sichuanensis	HSAUP II.2650-1 T	AB453881	–	–		
C. siddiquii	CBS 196.62 T	MN688823	MN688850	–		
C. siddiquii	CBS 142.78	MN688824	MN688851	–		
C. soli	CBS 222.96 T	KY905679	KY905691	KY905698		
C. sorghina	BRIP 15900 T	KJ415558	KJ415388	KJ415435		
C. spicifera	CBS 274.52	JN192387	JN600979	JN601023		
C. sporobolicola	BRIP 23040b T	MH414908	MH433652	MH433671		
C. subpapendorfii	CBS 656.74 T	KJ909777	KM061791	KM196585		
C. thailandica	MFLUCC 15-0747 T	MH275057	MH412749	MH412764		
C. tribuli	CBS 126975 T	MN688825	MN688852	MN688875		
C. trifolii	CBS 173.55	HG779023	HG779124	–		
C. tripogonis	BRIP 12375 T	JN192388	JN600980	JN601025		
C. tropicalis	BRIP 14834 T	KJ415559	KJ415387	KJ415434		
C. tsudae	ATCC 44764 T	KC424596	KC747745	KC503940		
C. tuberculata	CBS 146.63 T	JX256433	JX276445	JX266599		
C. uncinata	CBS 221.52 T	HG779024	HG779134	–		
C. variabilis	CPC 28815 T	MF490822	MF490844	MF490865		
C. verrucosa	CBS 422.93	MN688826	MN688853	MN688876		
C. verruculosa	CBS 150.63	KP400652	KP645346	KP735695		
C. warraberensis	BRIP 14817 T	MH414909	MH433653	MH433672		

C. xishuangbannaensis	MFLUCC 17-2271 T	MH275058	MH412750	MH412765		
Bipolaris maydis	CBS 136.29 T	KJ909769	KM034845	KM093793		
Johnalcornia aberrans	CBS 510.91 T	KJ415522	KJ415424	KJ415473		
Pyrenophora poae	BRIP 10953	KJ415566	KJ415380	KJ415427		
**Species**	**Culture collection number**	**ITS**	***tef***	***tub2***	***cal***	***his***
Diaporthe acaciarum	CBS 138862 T	KP004460		KP004509		KP004504
D. amaranthophila	MAFF 246900 T	LC459575	LC459577	LC459579	LC459583	LC459581
D. amaranthophila	MAFF 246901	LC459576	LC459578	LC459580	LC459584	LC459582
D. ambigua	CBS 114015 T	KC343010	KC343736	KC343978	KC343252	KC343494
D. angelicae	CBS 111592 T	KC343027	KC343753	KC343995	KC343269	KC343511
D. batatas	CBS 122.21 T	KC343040	KC343766	KC344008	KC343282	KC343524
D. beilharziae	BRIP 54792 T	JX862529	JX862535	KF170921		
D. biguttulata	CFCC 52584	MH121519	MH121561	MH121598	MH121437	MH121477
D. biguttulata	ZJUD47 T	KJ490582	KJ490461	KJ490403		KJ490524
D. brasiliensis	CBS 133183 T	KC343042	KC343768	KC344010	KC343284	KC343526
D. brasiliensis	LGMF926	KC343043	KC343769	KC344011	KC343285	KC343527
D. caatingaensis	CBS 141542 T	KY085927	KY115603	KY115600		KY115605
D. caatingaensis	URM7484	KY085928		KY115602	KY115599	KY115606
D. caryae	CFCC 52563 T	MH121498	MH121540	MH121580	MH121422	MH121458
D. caryae	CFCC 52564	MH121499	MH121541	MH121581	MH121423	MH121459
D. chiangraiensis	MFLUCC 17-1670	MF190118	MF377599			
D. chiangraiensis	MFLUCC 17-1669 T	MF190119	MF377598			
D. cichorii	MFLUCC 17-1023 T	KY964220	KY964176	KY964104	KY964133	
D. cinnamomi	CFCC 52569 T	MH121504	MH121546	MH121586		MH121464

D. cinnamomi	CFCC 52570	MH121505	MH121547	MH121587		MH121465
D. citriasiana	CGMCC 3.15224	JQ954645	JQ954663	KC357459	KC357491	KJ490515
D. convolvuli	CBS 124654 [T]	KC343054	KC343780	KC344022	KC343296	KC343538
D. cucurbitae	CBS 136.25	KC343031	KC343757	KC343999	KC343273	KC343515
D. cucurbitae	DAOM 42078 [T]	KM453210	KM453211	KP118848		KM453212
D. cuppatea	CBS 117499 [T]	KC343057	KC343783	KC344025	KC343299	KC343541
D. cyatheae	YMJ 1364 [T]	JX570889	KC465406	KC465403	KC465410	
D. discoidispora	ZJUD89 [T]	KJ490624	KJ490503	KJ490445		KJ490566
D. discoidispora	ZJUD87	KJ490622	KJ490501	KJ490443		KJ490564
D. ganjae	CBS 180.91 [T]	KC343112	KC343838	KC344080	KC343354	
D. ganjae	PSCG489	MK626955	MK654897	MK691287	MK691202	MK726204
D. goulteri	BRIP 55657a [T]	KJ197290	KJ197252	KJ197270		
D. gulyae	BRIP 54025 [T]	JF431299	JN645803	KJ197271		
D. gulyae	MFLUCC 17-1026	KY964223	KY964179	KY964107	KY964136	
D. helianthi	CBS 592.81 [T]	KC343115	KC343841	KC344083	KC343357	KC343599
D. hordei	CBS 481.92 [T]	KC343120	KC343846	KC344088	KC343362	KC343604
D. infecunda	CBS 133812 [T]	KC343126	KC343852	KC344094	KC343368	KC343610
D. infecunda	CPC 20293	KC343129	KC343855	KC344097	KC343371	KC343613
D. infertilis	CBS 230.52 [T]	KC343052	KC343778	KC344020	KC343294	KC343536
D. infertilis	CPC 20322	KC343053	KC343779	KC344021	KC343295	KC343537
D. leucospermi	CBS 111980 [T]	JN712460	KY435632	KY435673	KY435663	KY435653
D. longispora	CBS 194.36 [T]	KC343135	KC343861	KC344103	KC343377	KC343619
D. lusitanicae	CBS 123212	KC343136	KC343862	KC344104	KC343378	KC343620
D. lusitanicae	CBS 123213 [T]	MH863280	KC343863	KC344105	KC343379	KC343621
D. megalospora	CBS 143.27 [T]	KC343140	KC343866	KC344108	KC343382	KC343624

D. melonis	CBS 507.78 ^{T}	KC343142	KC343868	KC344110	KC343384	KC343626
D. melonis	SoyYS1712	LC360101	LC377213	LC377206		
D. middletonii	BRIP 54884e ^{T}	KJ197286	KJ197248	KJ197266		
D. middletonii	BRIP 57329	KJ197285	KJ197247	KJ197265		
D. minusculata	CGMCC 3.20098 ^{T}	MT385957	MT424692	MT424712	MW022475	MW022499
D. minusculata	GZCC 19-0345	MT797184	MT793027	MT793038	MW022476	MW022500
D. myracrodruonis	URM 7972 ^{T}	MK205289	MK213408	MK205291	MK205290	
D. neoarctii	CBS 109490 ^{T}	KC343145	KC343871	KC344113	KC343387	KC343629
D. novem	PL42	JQ697843	JQ697856			
D. novem	CBS 127270 ^{T}	KC343156	KC343882	KC344124	KC343398	KC343640
D. ovalispora	CGMCC 3.17256 ^{T}	KJ490628	KJ490507	KJ490449		KJ490570
D. oxe	CBS 133186 ^{T}	KC343164	KC343890	KC344132	KC343406	KC343648
D. oxe	CBS 133187	KC343165	KC343891	KC344133	KC343407	KC343649
D. paranensis	CBS 133184 ^{T}	KC343171	KC343897	KC344139	KC343413	KC343655
D. paranensis	LMICRO417	KY461115	KY461116			
D. passiflorae	DJY16A1-5	MH595929	MH621353	MH621349		
D. phaseolorum	CBS 116019	KC343175	KC343901	KC344143	KC343417	KC343659
D. phaseolorum	AR4203 ^{T}	KJ590738	KJ590739	KJ610893	KJ612135	KJ659220
D. racemosae	CPC 26646 ^{T}	MG600223	MG600225	MG600227	MG600219	MG600221
D. raonikayaporum	MFLUCC 14-1136	KU712449	KU749369	KU743988	KU749356	
D. raonikayaporum	CBS 133182 ^{T}	KC343188	KC343914	KC344156	KC343430	KC343672
D. rosae	MFLUCC 17-2658 ^{T}	MG828894		MG843878	MG829273	
D. rosae	MFLUCC 17-2574	MG906793	MG968954	MG968952		
D. rosiphthora	COAD 2913	MT311196	MT313692		MT313690	
D. rosiphthora	COAD 2914 ^{T}	MT311197	MT313693		MT313691	

D. sackstonii	BRIP 54669b T	KJ197287	KJ197249	KJ197267		
D. schini	CBS 133181 T	KC343191	KC343917	KC344159	KC343433	KC343675
D. schini	CPC 20286	KC343192	KC343918	KC344160	KC343434	KC343676
D. schoeni	MFLU 15-1279 T	KY964226	KY964182	KY964109	KY964139	
D. schoeni	MFLU 15-2609	KY964229	KY964185	KY964112	KY964141	
D. sclerotioides	CBS 296.67 T	KC343193	KC343919	KC344161	KC343435	KC343677
D. sclerotioides	CBS 710.76	KC343194	KC343920	KC344162	KC343436	KC343678
D. serafiniae	BRIP 55665a T	KJ197274	KJ197236	KJ197254		
D. serafiniae	BRIP 54136	KJ197273	KJ197235	KJ197253		
D. siamensis	MFLUCC 10-573a T	JQ619879	JX275393	JX275429	JX197422	
D. siamensis	MFLUCC 12-0300	KT459417	KT459451	KT459435	KT459467	
D. sojae	FAU635 T	KJ590719	KJ590762	KJ610875	KJ612116	KJ659208
D. sojae	FAU636	KJ590718	KJ590761	KJ610874	KJ612115	KJ659207
D. stewartii	CBS 193.36 T	MH867279	GQ250324	JX275421	JX197415	
D. stewartii	MN1	KX668416	KX852355			
D. subellipicola	KUMCC 17-0153 T	MG746632	MG746633	MG746634		
D. subordinaria	CBS 464.90 T	KC343214	KC343940	KC344182	KC343456	KC343698
D. subordinaria	CBS 101711	KC343213	KC343939	KC344181	KC343455	KC343697
D. tarchonanthi	CBS 146073 T	MT223794		MT223733		MT223759
D. tecomae	CBS 100547 T	KC343215	KC343941	KC344183	KC343457	KC343699
D. tectonendophytica	MFLUCC 13-0471 T	NR_147591	KU749367	KU743986	KU749354	
D. tectonendophytica	LC8115	KY491550	KY491560	KY491570		
D. terebinthifolii	CBS 133180 T	KC343216	KC343942	KC344184	KC343458	KC343700
D. terebinthifolii	CPC 20285	KC343218	KC343944	KC344186	KC343460	KC343702
D. thunbergiicola	MFLUCC 12-0033 T	KP715097	KP715098			

D. tulliensis	BRIP 62248a ^{T}	KR936130	KR936133	KR936132		
D. tulliensis	RS^{T}-1	MN911382	MT121969			
D. unshiuensis	CGMCC 3.17566	KJ490584	KJ490463	KJ490405		KJ490526
D. unshiuensis	ZJUD50	KJ490585	KJ490464	KJ490406		KJ490527
D. unshiuensis	ZJUD52 ^{T}	KJ490587	KJ490466	KJ490408		KJ490529
D. unshiuensis	PSCG339	MK626928	MK654879	MK691300	MK691181	MK726188
D. unshiuensis	ZHKUCC 22-0067	ON322895		ON315085	ON315001	ON315025
D. unshiuensis	JZB320309	PQ569052	PQ573060	PQ573062	PQ573064	PQ573066
D. unshiuensis	JZB320310	PQ569053	PQ573061	PQ573063	PQ573065	PQ573067
D. vexans	CBS 127.14	KC343229	KC343955	KC344197	KC343471	KC343713
D. vexans	FAU597	KJ590734	KJ590774	KJ610889	KJ612131	KJ659216
D. vochysiae	LGMF1583 ^{T}	MG976391	MK007526	MK007527	MK007528	MK033323
D. yunnanensis	CGMCC 3.18289 ^{T}	KX986796	KX999188	KX999228	KX999290	
D. yunnanensis	LC8107	KY491542	KY491552	KY491562	KY491572	
D. amygdali	CBS 126679 ^{T}	KC343022	KC343748	KC343990	KC343264	KC343506
D. amygdali	CBS 115620	KC343020	KC343746	KC343988	KC343262	KC343504

Ex-type, neo-type and epi-type cultures are marked with superscript T and sequences generated in the present study are in bold.
